# Glomus tumor in the floor of the mouth: a case report and review of the literature

**DOI:** 10.1186/s12957-018-1503-6

**Published:** 2018-10-10

**Authors:** Haixiao Zou, Li Song, Mengqi Jia, Li Wang, Yanfang Sun

**Affiliations:** 1 Department of Stomatology, The Second Affiliated Hospital of Nanchang University, Nanchang, China; 2 State Key Laboratory Breeding Base of Basic Science of Stomatology (Hubei-MOST) and Key Laboratory of Oral Biomedicine Ministry of Education, School and Hospital of Stomatology, Wuhan University, Wuhan, China; 3 Department of Oral and Maxillofacial Surgery, School and Hospital of Stomatology, Wuhan University, No. 237 Luoyu Street, Wuhan, 430079 Hubei China; 4 Department of Pathology, School and Hospital of Stomatology, Wuhan University, Wuhan, China

**Keywords:** Glomus tumor, Floor of mouth, Oral surgery

## Abstract

**Background:**

Glomus tumors are rare benign neoplasms that usually occur in the upper and lower extremities. Oral cavity involvement is exceptionally rare, with only a few cases reported to date.

**Case presentation:**

A 24-year-old woman with complaints of swelling in the left floor of her mouth for 6 months was referred to our institution. Her swallowing function was slightly affected; however, she did not have pain or tongue paralysis. Enhanced computed tomography revealed a 2.8 × 1.8 × 2.1 cm-sized well-defined, solid, heterogeneous nodule above the mylohyoid muscle. The mandible appeared to be uninvolved. The patient underwent surgery via an intraoral approach; histopathological examination revealed a glomus tumor. The patient has had no evidence of recurrence over 4 years of follow-up.

**Conclusions:**

Glomus tumors should be considered when patients present with painless nodules in the floor of the mouth.

## Background

The glomus body is a special arteriovenous anastomosis and functions in thermal regulation. Glomus tumors are rare, benign, mesenchymal tumors that originate from modified smooth muscle cells of the normal glomus body [1]. Glomus tumors account for only 1.6% of all soft tissue tumors and typically present as blue-red nodules (sized < 1 cm) that occur in the deep dermis or subcutis region [2]. These tumors are relatively common in the upper and lower extremities, particularly in the subungual site, but rarely occur in mucinous regions or the viscera [3]. Oral cavity involvement is exceptionally rare, with very few cases having been reported to date. Here, we present a case of an unusual glomus tumor that originated from the left floor of the mouth.

## Case presentation

A 24-year-old woman with a 6-month history of swelling in the left floor of her mouth was referred to our institution. Although she experienced slight difficulty in swallowing, she did not experience pain or tongue paralysis. Her medical and family histories were unremarkable.

**Fig. 1:**
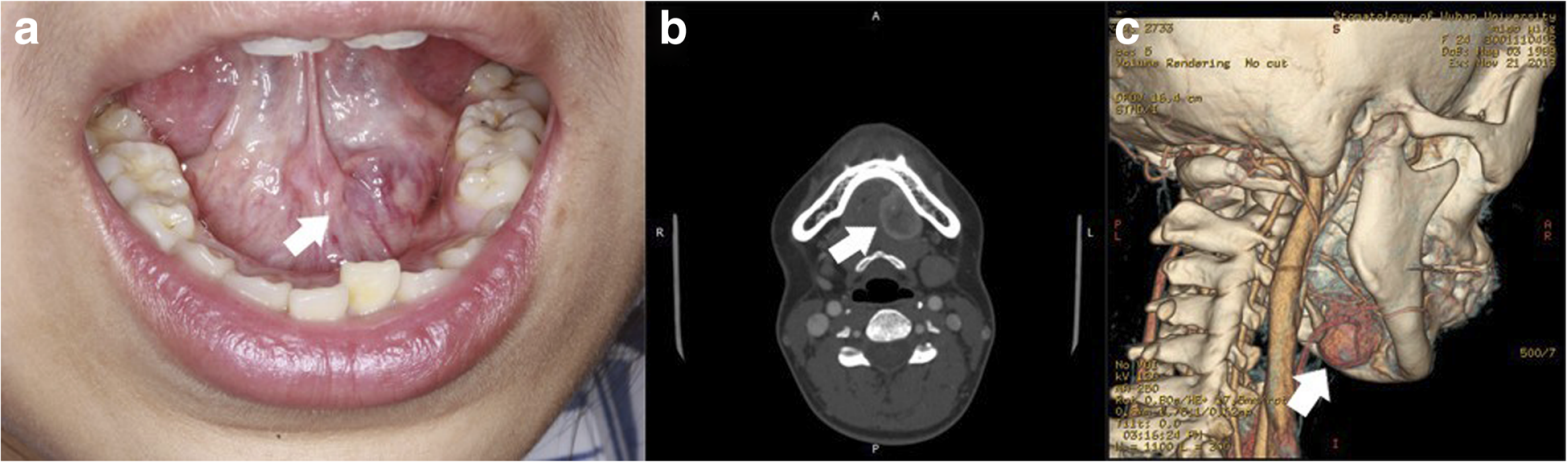
Physical examination and computed tomography findings. **a** A light bluish, solid neoplastic lesion measuring 3 cm in size was palpated in the left floor of the mouth, and the covered mucosa had no ulcers (arrow). **b** A computed tomography scan of the head and neck shows a well-defined, solid, heterogeneous enhanced nodule in the left floor of the mouth. There was no obvious infiltration in the surrounding soft tissue, and the lingual cortical bone of mandible was not involved (arrow). **c** A three-dimensional image showing the lesion was located lateral to the hyoid bone and adjacent to the left sublingual fossa of the mandible with abundant blood flow (arrow)

Intraoral examination revealed a well-defined 3.5 × 3 × 2 cm-sized solid, spherical submucosal nodule adjacent to the sublingual gland; the nodule was covered with light bluish smooth mucus (Fig. 1a). The patient experienced slight pain when pressure was applied to the tumor. Mobility and sensory functions of the tongue were normal, and no lymphadenopathy in the submandibular region was detected on palpation. All relevant laboratory test results were normal. Enhanced computed tomography revealed a 2.8 × 1.8 × 2.1 cm-sized well-defined, solid, heterogeneous nodule that did not appear to involve the mandible (Fig. 1b). In addition, a three-dimensionally reconstructed image showed a nodular lesion occupying the left floor of the mouth with abundant blood flow (Fig. 1c). No enlarged lymph nodes were found in the submental or submandibular regions.

**Fig. 2:**
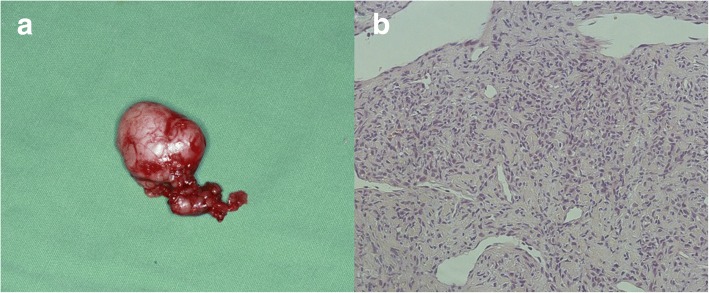
Macroscopic and histopathological examinations of the resected specimen (hematoxylin and eosin staining, magnification × 200). **a** The resected specimen was grayish white, with a diameter of approximately 3 cm, and was soft, compressible, covered with capsule, and had a firm attachment to the sublingual gland. **b** Tumor cells were round, oval, polyhedral, or fusiform arranged in organoid and sheet-like patterns with vascular lumens. Nuclei were round or ovoid with eosinophilic cytoplasm and had no obvious atypical and active mitotic activity. Cell borders were not clearly delineated

The initial clinical impression was a benign salivary gland tumor, dermoid cyst, or benign connective tissue neoplasm. The patient was scheduled for surgery via an intraoral approach. First, an elliptical incision was made around the periphery of the sublingual gland through only the oral mucosa, and a full-thickness tissue flap was prepared along the lingual aspect of the sublingual gland. After the sublingual gland was freed from its surrounding tissue with blunt dissection, a well-circumscribed tumor without capsular extension was found beneath the body of the sublingual gland and located above the submandibular gland duct and lingual nerve. The submandibular gland duct and lingual nerve were carefully freed from the tumor surface, and the complete tumor was excised along with the sublingual gland (Fig. 2a). The tissue sample was fixed with 10% formalin and submitted for histopathological diagnosis.

Microscopically, the tumor cells were round, oval, polyhedral, or fusiform and were arranged in organoid and sheet-like patterns with vascular lumens. Most of their nuclei were small and round within an amphiphilic or slightly eosinophilic cytoplasm. Nuclear atypia was rare (Fig. 2b).

Immunohistochemistry revealed that the tumor cells yield positive results for vimentin and alpha-smooth muscle actin, but negative results for desmin, anti-cytokeratin (AE)1 or AE3, cluster of differentiation (CD)31 and CD34, and S-100, and exhibited a Ki-67 index of 5%. These findings were consistent with those for a glomus tumor.

After surgery, the patient had an uneventful recovery with primary healing and had no evidence of recurrence over 4 years of follow-up.

## Discussion and conclusions

Glomus tumors are rare mesenchymal tumors that occur due to glomus body hyperplasia or hamartomatous development, and they appear to originate from modified smooth muscle cells [1]. These tumors are categorized into three types based on their histological appearances: glomangiomas (20% of cases), solid glomus tumors (75%), and glomangiomyomas (5%) [4]. In our patient, the solid-type tumor was predominant.

Such tumors mainly arise in the dermis or subcutaneous tissues of the hands and feet, especially the tips of fingers and toes. Extradigital glomus tumors are rare; fewer than 1% of glomus tumors are found in the head region [5], and a lesion located in the floor of the mouth has not been reported previously. Recently published case reports regarding glomus tumors of the oral cavity were reviewed [3, 6–39]. The patient characteristics of all 37 cases (including our case) are shown in Table 1. The neoplasms developed in 23 women and 14 men (male to female, 1.6:1), and patient age ranged from 10 to 85 years (median, 52 years). In most cases, the tumors were located on the lip (*n* = 14, lower 4, upper 10), followed by the buccal mucosa (*n* = 7), tongue (*n* = 5), hard palate (*n* = 6), gingiva (*n* = 2), maxilla (*n* = 1), the floor of the mouth (*n* = 1), and multiple locations (*n* = 1). Unfortunately, many of the cases documented had an unknown clinical presentation or medical history. Due to inadequate figures or illustrations in these articles, the histologic types of the tumors were not included in our table. Of the cases with available information, the size of the glomus tumor ranged from 0.3 to 4.5 cm. Some lesions were painful, and most were asymptomatic. All lesions were completely excised. Follow-up information was only available in 11 cases ranging from 2 months to 7 years; local recurrence was noted in two cases. Owing to the low incidence rate of glomus tumors in the head and neck region, accurate information on the peak incidence period and sex ratio remain unclear.

**Table 1:** Clinical treatment and outcome features of cases of glomus tumors in the oral cavity

Case	Authors	Year	Gender	Age(years)	Site	Size(cm)	Clinical presentation	Treatment	Follow-up time	Outcome
1	Langer [6]	1949	M	52	Hard palate	NA	NA	NA	NA	NA
2	King [7]	1954	M	32	Gingiva	NA	No symptoms	NA	NA	NA
3	Kirschner and Strassburg [8]	1962	M	56	Gingiva/alveolar mucosa	NA	NA	NA	NA	NA
4	Frenkel [9]	1965	M	13	Buccal mucosa	NA	NA	NA	NA	NA
5	Harris and Griffin [10]	1965	F	35	Maxilla	0.5 × 0.25 cm	Pain	Surgery	2 years	NED
6	Sidhu [11]	1967	F	10	Hard palate	NA	Painless neoplasm	NA	NA	NA
7	Charles [12]	1976	F	17	Hard palate	NA	NA	NA	NA	NA
8	Lele [13]	1977	M	35	Hard palate	1.5 × 1 cm	Painless neoplasm	Surgery	6 months	NED
9	Sato et al. [14]	1979	M	29	Tongue	NA	Painless neoplasm	NA	NA	NA
10	Tajima et al. [15]	1981	F	63	Tongue	NA	Painless neoplasm	NA	NA	NA
11	Saku et al. [16]	1985	M	45	Buccal mucosa	4.5 × 3 × 3.5 cm	No symptoms	Surgery	NA	NA
12	Ficarra et al. [3]	1986	F	51	Upper lip	NA	No symptoms	NA	NA	NA
13	Moody et al. [17]	1986	F	65	Upper lip	1 × 0.5 × 0.5 cm	No symptoms	Surgery	NA	NA
14	Stajcic and Bojic [18]	1987	M	55	Tongue	NA	Painless neoplasm	Surgery	NA	NA
15	Geraghty et al. [19]	1992	M	71	Hard palate	1.5 cm	No symptoms	Surgery	NA	NA
16	Kusama et al. [20]	1995	M	57	Upper lip	NA	Painless swelling	Surgery	4 years	NED
17	Savaci et al. [22]	1996	M	55	Buccal mucosa	1 cm	Pain	Surgery	NA	NA
18	Sakashita et al. [23]	1997	M	54	Upper lip	1.2 × 1 cm	Painless swelling	Surgery	NA	NA
19	Yu et al. [24]	2000	F	54	Left mandibular area, lip, anterior buccal mucosa	NA	Painless neoplasm	Surgery	NA	NA
20	Kessaris et al. [25]	2001	F	46	Hard palate	1.8 cm	Painless swelling	Surgery	3 years	NED
21	Rallis et al. [27]	2004	F	85	Upper lip	1.3 × 1 × 1 cm	Painful swelling	Surgery	1.5 years	NED
22	Quesada et al. [26]	2004	M	61	Tongue	3 cm	Painless neoplasm	Surgery	7 years	Recurrence
23	Lanza et al. [28]	2005	M	65	Lower lip	NA	Painful mass	NA	NA	NA
24	Ide et al. [30]	2008	M	57	Upper lip	0.8 cm	NA	Surgery	NA	NA
25	Ide et al. [30]	2008	M	54	Upper lip	1.2 cm	NA	Surgery	NA	NA
26	Wang et al. [31]	2008	M	51	Buccal mucosa	NA	NA	Surgery	NA	NA
27	Wang et al. [31]	2008	F	58	Buccal mucosa	NA	NA	Surgery	NA	NA
28	Boros et al. [32]	2010	M	34	Lower lip	NA	NA	Surgery	NA	NA
29	Yoruk et al. [34]	2010	F	30	Buccal mucosa	2 × 1.1 × 0.5 cm	Painless neoplasm	Surgery	1 years	NED
30	Dérand et al. [33]	2010	F	11	Lower lip	0.3 cm	No symptoms	Surgery	7 years	NED
31	Veros et al. [35]	2012	F	24	Buccal mucosa	1 × 1 cm	Painful mass	Surgery	2 months	Recurrence
32	Chou et al. [36]	2015	M	39	Upper lip	NA	NA	NA	NA	NA
33	Monaghan [37]	2017	M	73	Upper lip	1 cm	No symptoms	Surgery	NA	NA
34	Vasconcelos et al. [39]	2018	M	67	Upper lip	1 cm	Painful swelling	Surgery	3.3 years	NED
35	Smith et al. [38]	2018	M	26	Lower lip	1.5 × 0.5 × 0.5 cm	Painful mass	Surgery	NA	NA
36	Smith et al. [38]	2018	F	58	Tongue	2 × 1 cm	No symptoms	Surgery	NA	NA
37	Our case		M	24	The floor of the mouth	2.8 × 1.8 × 2.1 cm	Painful swelling	Surgery	4 years	NED

*F* female, *M* male, *NA* not available, *NED* no evidence of death

Glomus tumors in the dermis or subcutaneous tissues of the hands and feet are usually < 1 cm in size [21]. However, glomus tumors in the head and neck region are larger, with average diameters of 1–1.5 cm [5]. There is no evidence that the tumor volume influences the patients’ prognoses [25]. Although cases of malignant glomus tumors have been documented, malignancies in the head and neck region are very rare [29], and no characteristic symptoms or imaging features have yet been reported. The diagnosis of malignant transformation of a glomus tumor still depends on its pathological examination. Tumors > 2 cm in size with atypical mitotic figures, moderate-to-high nuclear grade, and > 5 mitotic figures per 50 high-power fields are considered highly suspicious for malignancy [40]. A recent study found that *BRAF* V600E mutations may be associated with a malignant phenotype in glomus tumors [41]; however, larger cohorts and multicenter studies are required to confirm this finding.

Atypical performance may be the main reason why patients with head and neck glomus tumors postpone visiting the maxillofacial surgery clinic. Although cold sensitivity, spontaneous intermittent pain, and pinpoint tenderness are hallmarks of extraoral glomus tumors, few patients with oral glomus tumors who are referred to the maxillofacial clinic have these symptoms [32]. The lack of such sensations may be attributable to the varying distribution of nerve fibers in different anatomical regions; this notion remains to be explored.

The accurate preoperative diagnosis of intraoral glomus tumors remains challenging. Inaccurate diagnoses are largely attributed to this tumor’s rarity and the lack of distinguishing clinico-morphologic characteristics. Furthermore, such lesions have nonspecific and heterogeneous appearances on radiologic images. A glomus tumor may initially be diagnosed as a salivary tumor, sebaceous cyst, neurofibromatosis, dermoid cyst, teratoid tumor, vascular malformation, or another type of mesenchymal neoplasm [42]. Although vascular malformations and cystic soft tissue lesions can usually be ruled out using color duplex ultrasonography, the differential diagnosis of solid tumors remains challenging. Recently, ^18^fluorodopa (F-DOPA) positron emission tomography was used for detecting glomus tumors [43]; however, the validity and specificity of this technique for tumors in the head and neck region requires verification. As formal diagnostic guidelines are absent, histologic examination and immunohistochemical analysis remain the gold standards.

Histologically, the appearance of glomus tumors depends on their cellular compositions and differentiation levels. A typical solid glomus tumor is composed of small vascular channels surrounded by clusters of well-defined round cells with lightly eosinophilic cytoplasm, and a large central round or oval nucleus with no atypia. The immunohistochemical profile of glomus tumor cells includes positivity for vimentin, smooth muscle actin, and muscle-specific actin; moreover, positivity for desmin, CD34, and *BRAF* mutations has been identified in some cases [44]. Conversely, these tumors yield negative results for S-100, myoglobin, neurofilaments, and factor VIII-related antigen [27].

Glomus tumors should first be differentiated from tumors originating from the sublingual gland, where acinar and ductal structures can be observed histologically in such neoplasia [45]; these structures were not observed in our cases. Meanwhile, the sublingual gland is a common site for epithelial tumors; we found that epithelial markers were negative in this case, indicating that the tumor was not a neoplasm of epithelial original.

Secondly, the differential diagnosis for glomus tumors includes vascular tumors such as hemangioma, hemangioendothelioma, epithelioid hemangioma, kaposiform angiodermatitis, reactive angioendotheliomatosis, and angiosarcoma [25]. These tumors can be easily excluded based on histomorphologic features and the expression of endothelial cell markers [46].

Glomus cells, myopericytes, vascular smooth muscle cells, and myofibroblasts are derivatives of pericytes [47]; therefore, glomus tumors should also be distinguished from the most common of the perivascular tumors, including myofibromas, glomangiopericytoma, and myopericytoma. These tumors share many histologic, immunophenotypic, and cytogenetic features, and it is difficult to distinguish them from one another solely by immunohistochemical examination [30]. However, myopericytoma can be differentiated from the glomus tumor based on concentric perivascular growth of spindle neoplastic cells [16]. Myofibromas have a biphasic zonation pattern with light staining fascicles to whorls of myofibroblastic cells, and dark-staining zones of polygonal cells associated with hemangiopericytoma-like vessels [48]. Glomus tumors are composed of cuboidal cells with distinct cell borders, and a round, centrally located nucleus, and they lack spindle cell morphology. The absence of both a multinodular and biphasic pattern would help to exclude myofibroma. Angioleiomyomas have a predominant vascular smooth muscle cell component. On histopathological examination, proliferation of vascular channels was noted, along with thick walls of circumferentially arranged spindle cells [32]. The histomorphological characteristics and positive expression of both actin and desmin can be used to positively identify a glomus tumor.

The majority of glomus tumors are entirely benign; hence, en bloc resection is an effective treatment. While incomplete resection may result in recurrence, local recurrence is very uncommon. Malignant glomus tumors are very rare and require multimodal integrated treatments [26].

We report the rare case of a glomus tumor in the floor of a patient’s mouth that showed no marked symptoms, which complicated its early diagnosis. Glomus tumors should be included in the initial differential diagnosis in patients presenting with painless nodules in the floor of the mouth. En bloc resection is an effective treatment, and patients should receive long-term counseling regarding the risk of recurrence.

## Abbreviations

AE1: Anti-cytokeratin 1
AE3: Anti-cytokeratin 3
CD: Cluster of differentiation
F-DOPA: ^18^Fluorodopa
